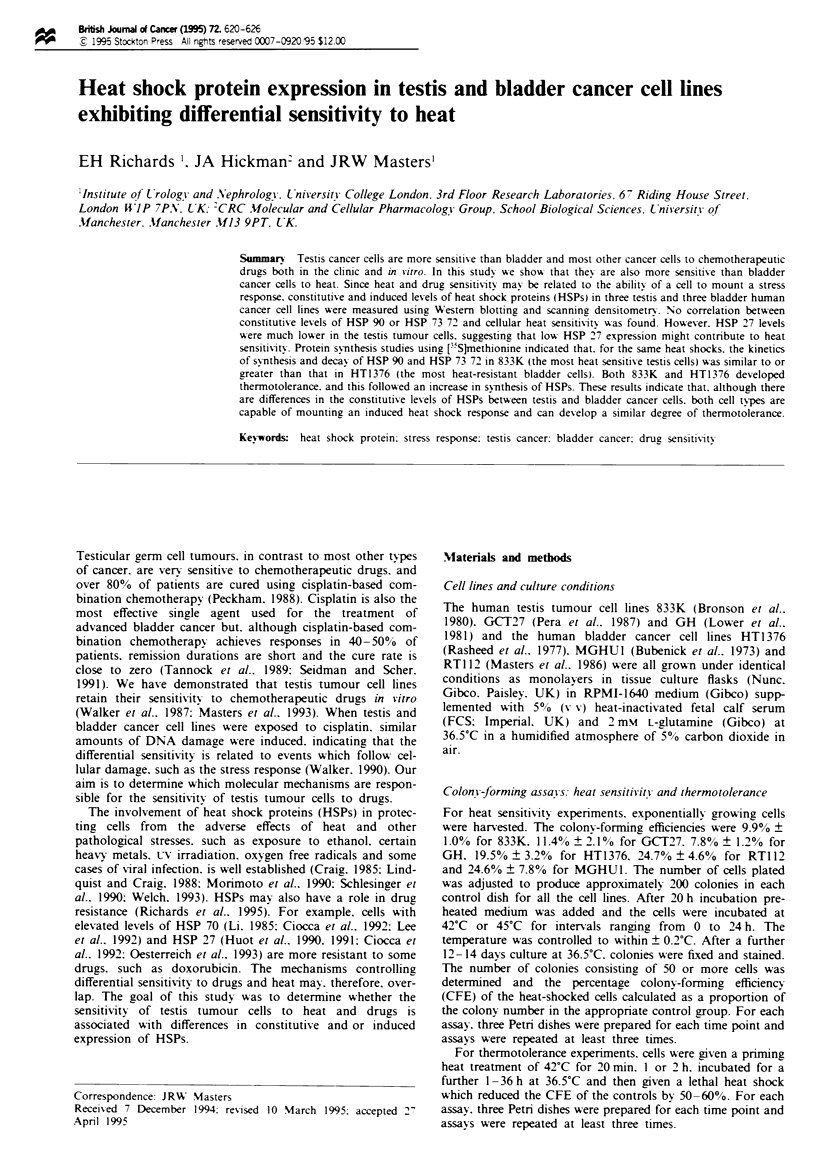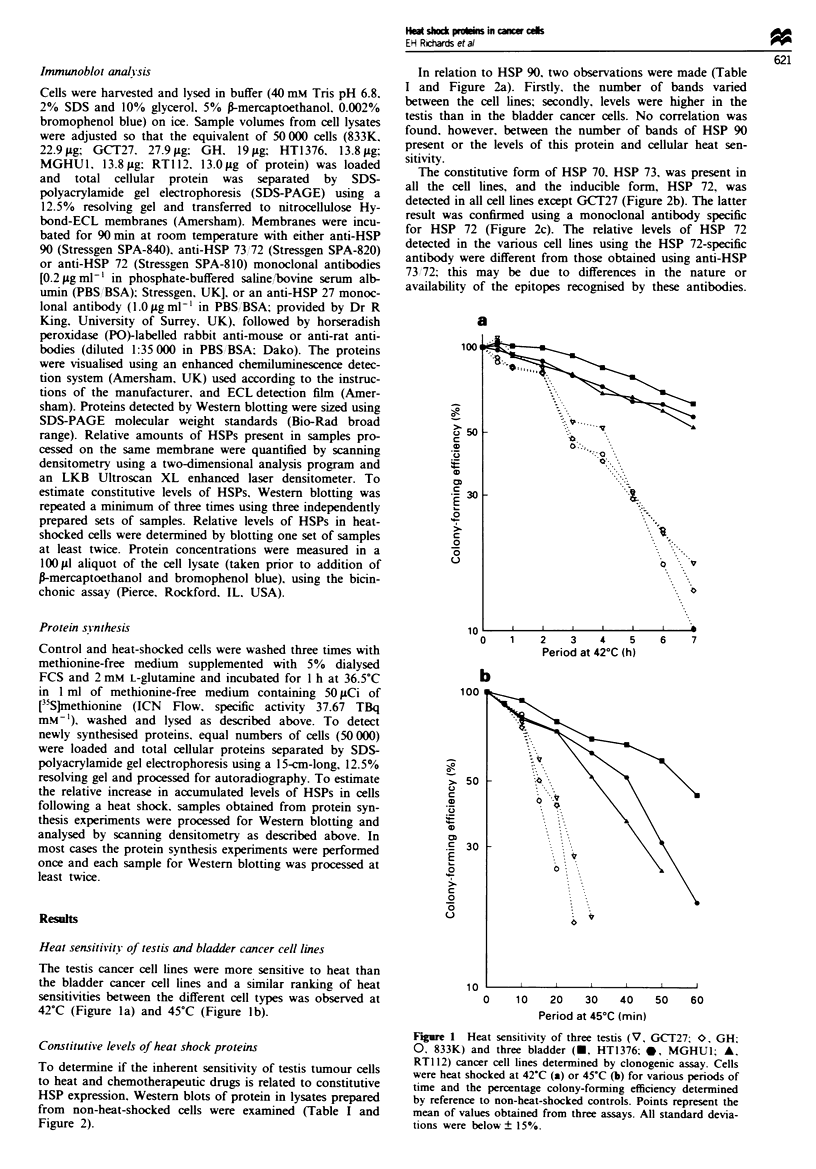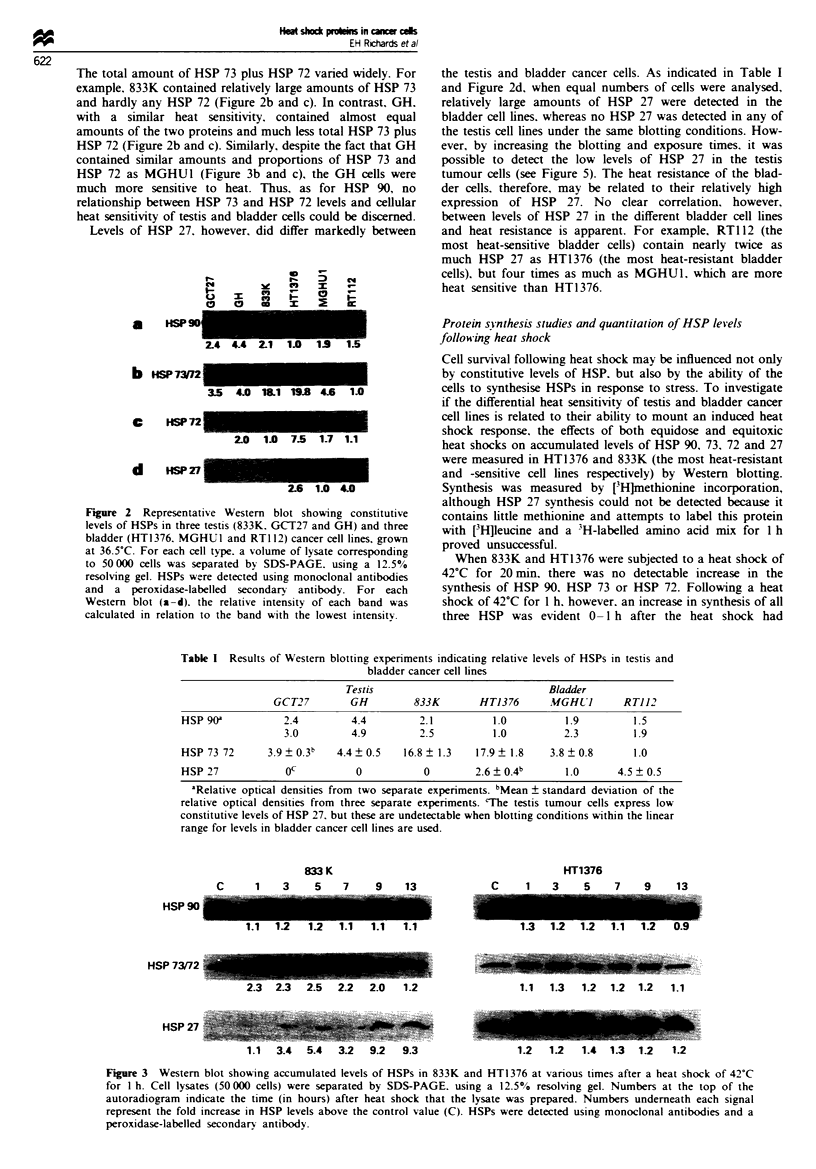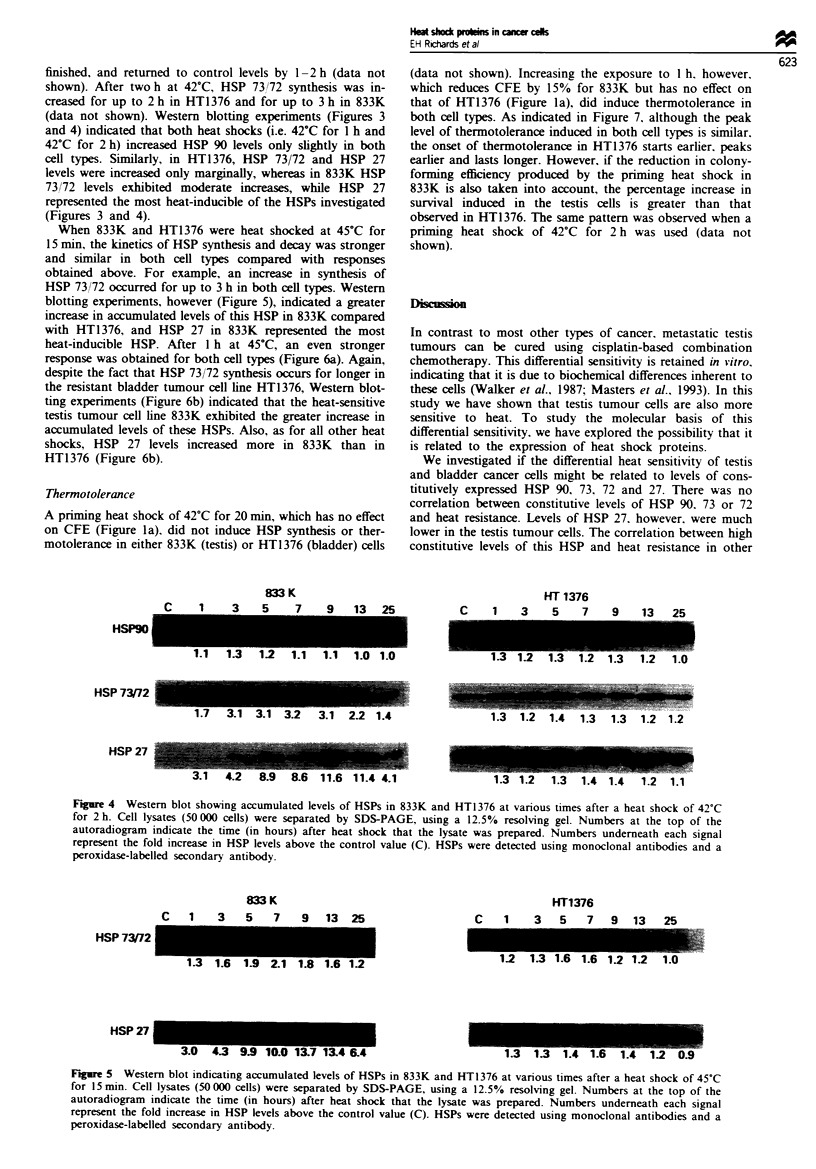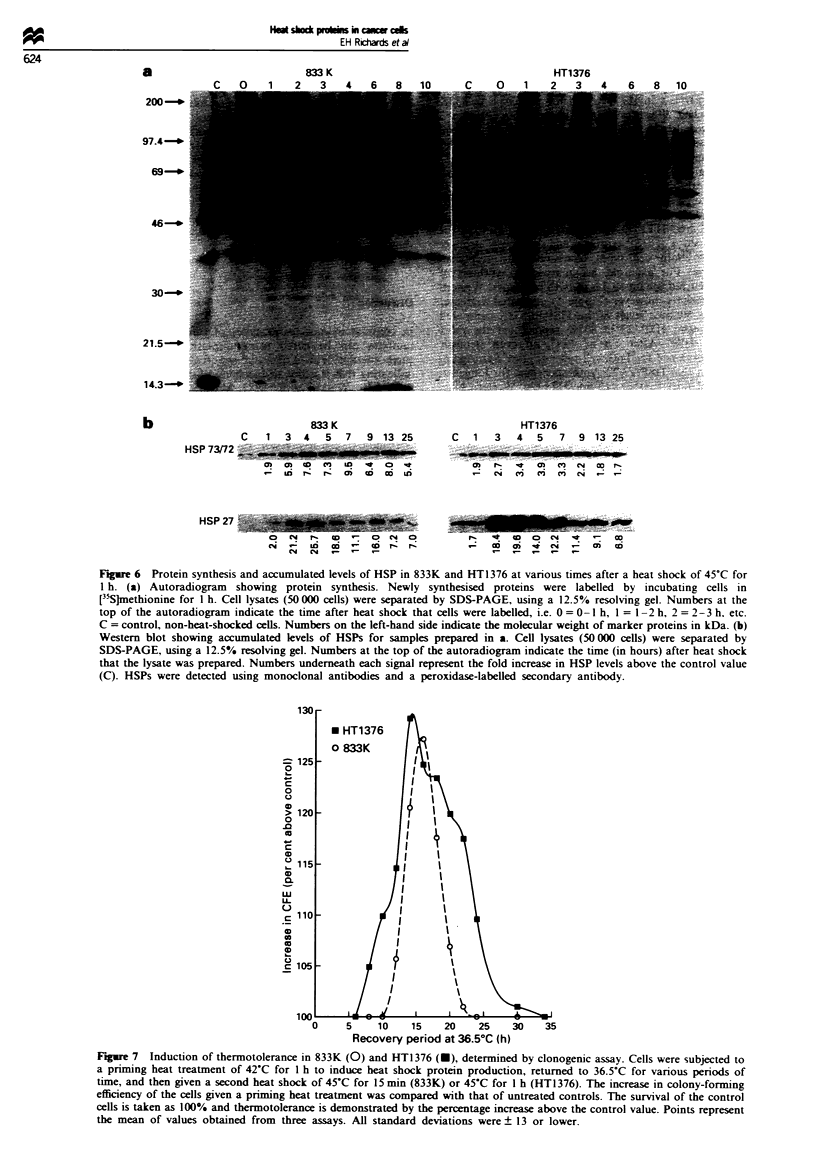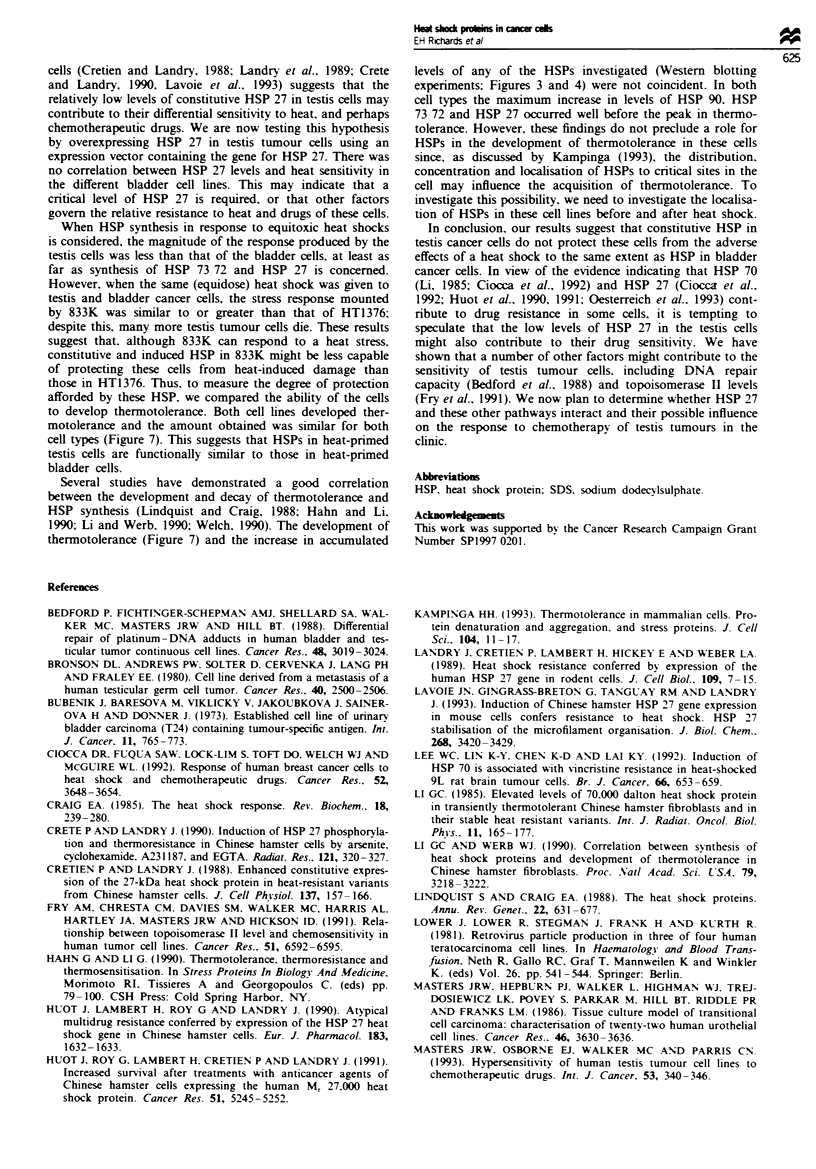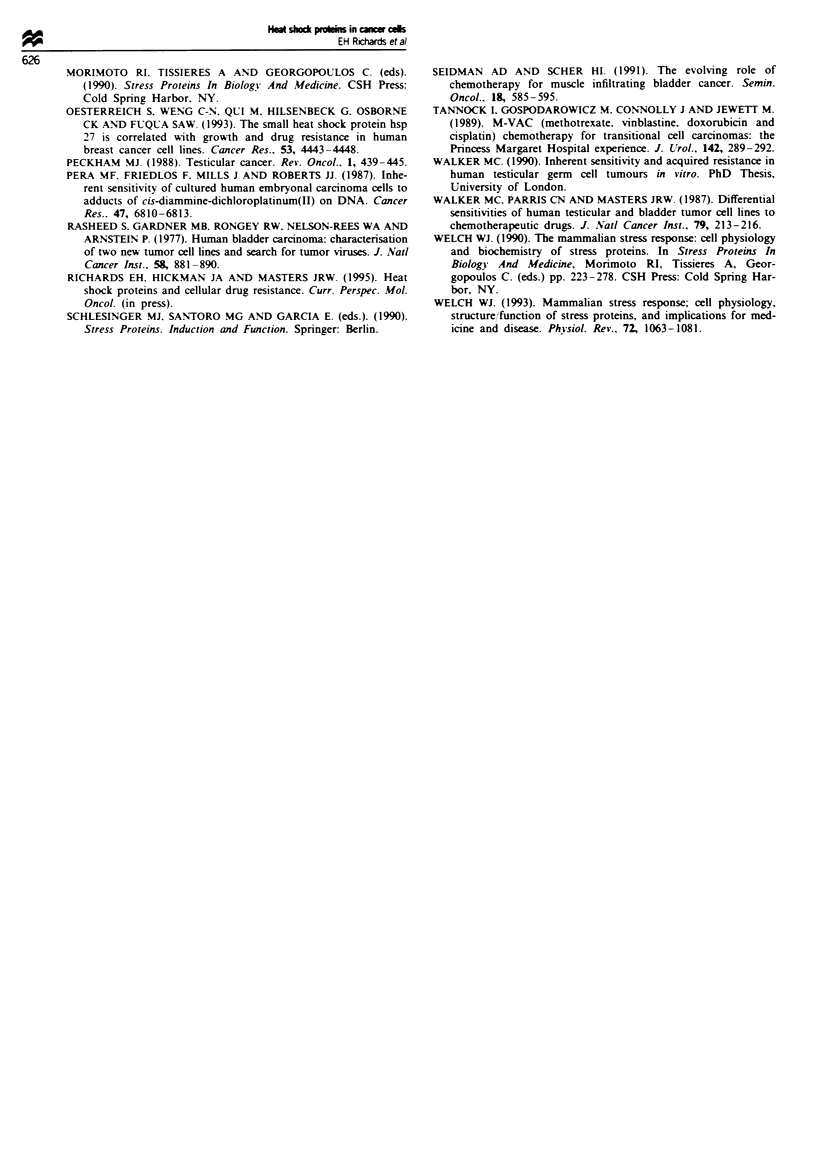# Heat shock protein expression in testis and bladder cancer cell lines exhibiting differential sensitivity to heat.

**DOI:** 10.1038/bjc.1995.383

**Published:** 1995-09

**Authors:** E. H. Richards, J. A. Hickman, J. R. Masters

**Affiliations:** Institute of Urology and Nephrology, University College London, UK.

## Abstract

**Images:**


British Journal of Cancer (1995) 72, 620-626

© 1995 Stockton Press All rights reserved 0007-0920/95 $12.00

### Heat shock protein expression in testis and bladder cancer cell lines exhibiting differential sensitivity to heat

EH Richards [1], JA Hickman [2] and JRW Masters [1]

[1]Institute of Urology and Nephrology, University College London, 3rd Floor Research Laboratories, 67 Riding House Street, London W1P 7PN, UK; [2]CRC Molecular and Cellular Pharmacology Group, School Biological Sciences, University of Manchester, Manchester M13 9PT, UK.

**Summary** Testis cancer cells are more sensitive than bladder and most other cancer cells to chemotherapeutic drugs both in the clinic and *in vitro*. In this study we show that they are also more sensitive than bladder cancer cells to heat. Since heat and drug sensitivity may be related to the ability of a cell to mount a stress response, constitutive and induced levels of heat shock proteins (HSPs) in three testis and three bladder human cancer cell lines were measured using Western blotting and scanning densitometry. No correlation between constitutive levels of HSP 90 or HSP 73/72 and cellular heat sensitivity was found. However, HSP 27 levels were much lower in the testis tumour cells, suggesting that low HSP 27 expression might contribute to heat sensitivity. Protein synthesis studies using [$^{35}$S]methionine indicated that, for the same heat shocks, the kinetics of synthesis and decay of HSP 90 and HSP 73/72 in 833K (the most heat sensitive testis cells) was similar to or greater than that in HT1376 (the most heat-resistant bladder cells). Both 833K and HT1376 developed thermotolerance, and this followed an increase in synthesis of HSPs. These results indicate that, although there are differences in the constitutive levels of HSPs between testis and bladder cancer cells, both cell types are capable of mounting an induced heat shock response and can develop a similar degree of thermotolerance.

**Keywords:** heat shock protein; stress response; testis cancer; bladder cancer; drug sensitivity

Testicular germ cell tumours, in contrast to most other types of cancer, are very sensitive to chemotherapeutic drugs, and over 80% of patients are cured using cisplatin-based combination chemotherapy (Peckham, 1988). Cisplatin is also the most effective single agent used for the treatment of advanced bladder cancer but, although cisplatin-based combination chemotherapy achieves responses in 40-50% of patients, remission durations are short and the cure rate is close to zero (Tannock *et al.*, 1989; Seidman and Scher, 1991). We have demonstrated that testis tumour cell lines retain their sensitivity to chemotherapeutic drugs *in vitro* (Walker *et al.*, 1987; Masters *et al.*, 1993). When testis and bladder cancer cell lines were exposed to cisplatin, similar amounts of DNA damage were induced, indicating that the differential sensitivity is related to events which follow cellular damage, such as the stress response (Walker, 1990). Our aim is to determine which molecular mechanisms are responsible for the sensitivity of testis tumour cells to drugs.

The involvement of heat shock proteins (HSPs) in protecting cells from the adverse effects of heat and other pathological stresses, such as exposure to ethanol, certain heavy metals, UV irradiation, oxygen free radicals and some cases of viral infection, is well established (Craig, 1985; Lindquist and Craig, 1988; Morimoto *et al.*, 1990; Schlesinger *et al.*, 1990; Welch, 1993). HSPs may also have a role in drug resistance (Richards *et al.*, 1995). For example, cells with elevated levels of HSP 70 (Li, 1985; Ciocca *et al.*, 1992; Lee *et al.*, 1992) and HSP 27 (Huot *et al.*, 1990, 1991; Ciocca *et al.*, 1992; Oesterreich *et al.*, 1993) are more resistant to some drugs, such as doxorubicin. The mechanisms controlling differential sensitivity to drugs and heat may, therefore, overlap. The goal of this study was to determine whether the sensitivity of testis tumour cells to heat and drugs is associated with differences in constitutive and/or induced expression of HSPs.

Correspondence: JRW Masters
Received 7 December 1994; revised 10 March 1995; accepted 27 April 1995

### Materials and methods

### Cell lines and culture conditions

The human testis tumour cell lines 833K (Bronson *et al.*, 1980), GCT27 (Pera *et al.*, 1987) and GH (Lower *et al.*, 1981) and the human bladder cancer cell lines HT1376 (Rasheed *et al.*, 1977), MGHU1 (Bubenick *et al.*, 1973) and RT112 (Masters *et al.*, 1986) were all grown under identical conditions as monolayers in tissue culture flasks (Nunc, Gibco, Paisley, UK) in RPMI-1640 medium (Gibco) supplemented with 5% (v/v) heat-inactivated fetal calf serum (FCS; Imperial, UK) and 2 mM L-glutamine (Gibco) at 36.5°C in a humidified atmosphere of 5% carbon dioxide in air.

### Colony-forming assays: heat sensitivity and thermotolerance

For heat sensitivity experiments, exponentially growing cells were harvested. The colony-forming efficiencies were 9.9% ± 1.0% for 833K, 11.4% ± 2.1% for GCT27, 7.8% ± 1.2% for GH, 19.5% ± 3.2% for HT1376, 24.7% ± 4.6% for RT112 and 24.6% ± 7.8% for MGHU1. The number of cells plated was adjusted to produce approximately 200 colonies in each control dish for all the cell lines. After 20 h incubation preheated medium was added and the cells were incubated at 42°C or 45°C for intervals ranging from 0 to 24 h. The temperature was controlled to within ± 0.2°C. After a further 12-14 days culture at 36.5°C, colonies were fixed and stained. The number of colonies consisting of 50 or more cells was determined and the percentage colony-forming efficiency (CFE) of the heat-shocked cells calculated as a proportion of the colony number in the appropriate control group. For each assay, three Petri dishes were prepared for each time point and assays were repeated at least three times.

For thermotolerance experiments, cells were given a priming heat treatment of 42°C for 20 min, 1 or 2 h, incubated for a further 1-36 h at 36.5°C and then given a lethal heat shock which reduced the CFE of the controls by 50-60%. For each assay, three Petri dishes were prepared for each time point and assays were repeated at least three times.

*Immunoblot analysis*

Cells were harvested and lysed in buffer (40 mM Tris pH 6.8, 2% SDS and 10% glycerol, 5% β-mercaptoethanol, 0.002% bromophenol blue) on ice. Sample volumes from cell lysates were adjusted so that the equivalent of 50 000 cells (833K, 22.9 μg; GCT27, 27.9 μg; GH, 19 μg; HT1376, 13.8 μg; MGHU1, 13.8 μg; RT112, 13.0 μg of protein) was loaded and total cellular protein was separated by SDS-polyacrylamide gel electrophoresis (SDS-PAGE) using a 12.5% resolving gel and transferred to nitrocellulose Hybond-ECL membranes (Amersham). Membranes were incubated for 90 min at room temperature with either anti-HSP 90 (Stressgen SPA-840), anti-HSP 73/72 (Stressgen SPA-820) or anti-HSP 72 (Stressgen SPA-810) monoclonal antibodies [0.2 $\mu g\ ml^{-1}$ in phosphate-buffered saline/bovine serum albumin (PBS/BSA); Stressgen, UK], or an anti-HSP 27 monoclonal antibody (1.0 $\mu g\ ml^{-1}$ in PBS/BSA; provided by Dr R King, University of Surrey, UK), followed by horseradish peroxidase (PO)-labelled rabbit anti-mouse or anti-rat antibodies (diluted 1:35 000 in PBS/BSA; Dako). The proteins were visualised using an enhanced chemiluminescence detection system (Amersham, UK) used according to the instructions of the manufacturer, and ECL detection film (Amersham). Proteins detected by Western blotting were sized using SDS-PAGE molecular weight standards (Bio-Rad broad range). Relative amounts of HSPs present in samples processed on the same membrane were quantified by scanning densitometry using a two-dimensional analysis program and an LKB Ultroscan XL enhanced laser densitometer. To estimate constitutive levels of HSPs, Western blotting was repeated a minimum of three times using three independently prepared sets of samples. Relative levels of HSPs in heat-shocked cells were determined by blotting one set of samples at least twice. Protein concentrations were measured in a 100 μl aliquot of the cell lysate (taken prior to addition of β-mercaptoethanol and bromophenol blue), using the bicinchonic assay (Pierce, Rockford, IL, USA).

*Protein synthesis*

Control and heat-shocked cells were washed three times with methionine-free medium supplemented with 5% dialysed FCS and 2 mM L-glutamine and incubated for 1 h at 36.5°C in 1 ml of methionine-free medium containing 50 μCi of [$^{35}$S]methionine (ICN Flow, specific activity 37.67 TBq $mM^{-1}$), washed and lysed as described above. To detect newly synthesised proteins, equal numbers of cells (50 000) were loaded and total cellular proteins separated by SDS-polyacrylamide gel electrophoresis using a 15-cm-long, 12.5% resolving gel and processed for autoradiography. To estimate the relative increase in accumulated levels of HSPs in cells following a heat shock, samples obtained from protein synthesis experiments were processed for Western blotting and analysed by scanning densitometry as described above. In most cases the protein synthesis experiments were performed once and each sample for Western blotting was processed at least twice.

### Results

*Heat sensitivity of testis and bladder cancer cell lines*

The testis cancer cell lines were more sensitive to heat than the bladder cancer cell lines and a similar ranking of heat sensitivities between the different cell types was observed at 42°C (Figure 1a) and 45°C (Figure 1b).

*Constitutive levels of heat shock proteins*

To determine if the inherent sensitivity of testis tumour cells to heat and chemotherapeutic drugs is related to constitutive HSP expression, Western blots of protein in lysates prepared from non-heat-shocked cells were examined (Table I and Figure 2).

In relation to HSP 90, two observations were made (Table I and Figure 2a). Firstly, the number of bands varied between the cell lines; secondly, levels were higher in the testis than in the bladder cancer cells. No correlation was found, however, between the number of bands of HSP 90 present or the levels of this protein and cellular heat sensitivity.

The constitutive form of HSP 70, HSP 73, was present in all the cell lines, and the inducible form, HSP 72, was detected in all cell lines except GCT27 (Figure 2b). The latter result was confirmed using a monoclonal antibody specific for HSP 72 (Figure 2c). The relative levels of HSP 72 detected in the various cell lines using the HSP 72-specific antibody were different from those obtained using anti-HSP 73/72; this may be due to differences in the nature or availability of the epitopes recognised by these antibodies.

**Figure 1** Heat sensitivity of three testis (▽, GCT27; ◇, GH; ○, 833K) and three bladder (■, HT1376; ●, MGHU1; ▲, RT112) cancer cell lines determined by clonogenic assay. Cells were heat shocked at 42°C (**a**) or 45°C (**b**) for various periods of time and the percentage colony-forming efficiency determined by reference to non-heat-shocked controls. Points represent the mean of values obtained from three assays. All standard deviations were below ± 15%.

The total amount of HSP 73 plus HSP 72 varied widely. For example, 833K contained relatively large amounts of HSP 73 and hardly any HSP 72 (Figure 2b and c). In contrast, GH, with a similar heat sensitivity, contained almost equal amounts of the two proteins and much less total HSP 73 plus HSP 72 (Figure 2b and c). Similarly, despite the fact that GH contained similar amounts and proportions of HSP 73 and HSP 72 as MGHU1 (Figure 3b and c), the GH cells were much more sensitive to heat. Thus, as for HSP 90, no relationship between HSP 73 and HSP 72 levels and cellular heat sensitivity of testis and bladder cells could be discerned.

Levels of HSP 27, however, did differ markedly between the testis and bladder cancer cells. As indicated in Table I and Figure 2d, when equal numbers of cells were analysed, relatively large amounts of HSP 27 were detected in the bladder cell lines, whereas no HSP 27 was detected in any of the testis cell lines under the same blotting conditions. However, by increasing the blotting and exposure times, it was possible to detect the low levels of HSP 27 in the testis tumour cells (see Figure 5). The heat resistance of the bladder cells, therefore, may be related to their relatively high expression of HSP 27. No clear correlation, however, between levels of HSP 27 in the different bladder cell lines and heat resistance is apparent. For example, RT112 (the most heat-sensitive bladder cells) contain nearly twice as much HSP 27 as HT1376 (the most heat-resistant bladder cells), but four times as much as MGHU1, which are more heat sensitive than HT1376.

**Figure 2** Representative Western blot showing constitutive levels of HSPs in three testis (833K, GCT27 and GH) and three bladder (HT1376, MGHU1 and RT112) cancer cell lines, grown at 36.5°C. For each cell type, a volume of lysate corresponding to 50 000 cells was separated by SDS-PAGE, using a 12.5% resolving gel. HSPs were detected using monoclonal antibodies and a peroxidase-labelled secondary antibody. For each Western blot (**a**–**d**), the relative intensity of each band was calculated in relation to the band with the lowest intensity.

*Protein synthesis studies and quantitation of HSP levels following heat shock*

Cell survival following heat shock may be influenced not only by constitutive levels of HSP, but also by the ability of the cells to synthesise HSPs in response to stress. To investigate if the differential heat sensitivity of testis and bladder cancer cell lines is related to their ability to mount an induced heat shock response, the effects of both equidose and equitoxic heat shocks on accumulated levels of HSP 90, 73, 72 and 27 were measured in HT1376 and 833K (the most heat-resistant and -sensitive cell lines respectively) by Western blotting. Synthesis was measured by [$^3$H]methionine incorporation, although HSP 27 synthesis could not be detected because it contains little methionine and attempts to label this protein with [$^3$H]leucine and a $^3$H-labelled amino acid mix for 1 h proved unsuccessful.

When 833K and HT1376 were subjected to a heat shock of 42°C for 20 min, there was no detectable increase in the synthesis of HSP 90, HSP 73 or HSP 72. Following a heat shock of 42°C for 1 h, however, an increase in synthesis of all three HSP was evident 0–1 h after the heat shock had

**Table I** Results of Western blotting experiments indicating relative levels of HSPs in testis and bladder cancer cell lines

| | *Testis* | | | *Bladder* | | |
|---|---|---|---|---|---|---|
| | *GCT27* | *GH* | *833K* | *HT1376* | *MGHU1* | *RT112* |
| HSP 90[a] | 2.4 | 4.4 | 2.1 | 1.0 | 1.9 | 1.5 |
| | 3.0 | 4.9 | 2.5 | 1.0 | 2.3 | 1.9 |
| HSP 73 72 | 3.9 ± 0.3[b] | 4.4 ± 0.5 | 16.8 ± 1.3 | 17.9 ± 1.8 | 3.8 ± 0.8 | 1.0 |
| HSP 27 | 0[C] | 0 | 0 | 2.6 ± 0.4[b] | 1.0 | 4.5 ± 0.5 |

[a]Relative optical densities from two separate experiments. [b]Mean ± standard deviation of the relative optical densities from three separate experiments. [c]The testis tumour cells express low constitutive levels of HSP 27, but these are undetectable when blotting conditions within the linear range for levels in bladder cancer cell lines are used.

**Figure 3** Western blot showing accumulated levels of HSPs in 833K and HT1376 at various times after a heat shock of 42°C for 1 h. Cell lysates (50 000 cells) were separated by SDS-PAGE, using a 12.5% resolving gel. Numbers at the top of the autoradiogram indicate the time (in hours) after heat shock that the lysate was prepared. Numbers underneath each signal represent the fold increase in HSP levels above the control value (C). HSPs were detected using monoclonal antibodies and a peroxidase-labelled secondary antibody.

finished, and returned to control levels by 1–2 h (data not shown). After two h at 42°C, HSP 73/72 synthesis was increased for up to 2 h in HT1376 and for up to 3 h in 833K (data not shown). Western blotting experiments (Figures 3 and 4) indicated that both heat shocks (i.e. 42°C for 1 h and 42°C for 2 h) increased HSP 90 levels only slightly in both cell types. Similarly, in HT1376, HSP 73/72 and HSP 27 levels were increased only marginally, whereas in 833K HSP 73/72 levels exhibited moderate increases, while HSP 27 represented the most heat-inducible of the HSPs investigated (Figures 3 and 4).

When 833K and HT1376 were heat shocked at 45°C for 15 min, the kinetics of HSP synthesis and decay was stronger and similar in both cell types compared with responses obtained above. For example, an increase in synthesis of HSP 73/72 occurred for up to 3 h in both cell types. Western blotting experiments, however (Figure 5), indicated a greater increase in accumulated levels of this HSP in 833K compared with HT1376, and HSP 27 in 833K represented the most heat-inducible HSP. After 1 h at 45°C, an even stronger response was obtained for both cell types (Figure 6a). Again, despite the fact that HSP 73/72 synthesis occurs for longer in the resistant bladder tumour cell line HT1376, Western blotting experiments (Figure 6b) indicated that the heat-sensitive testis tumour cell line 833K exhibited the greater increase in accumulated levels of these HSPs. Also, as for all other heat shocks, HSP 27 levels increased more in 833K than in HT1376 (Figure 6b).

### *Thermotolerance*

A priming heat shock of 42°C for 20 min, which has no effect on CFE (Figure 1a), did not induce HSP synthesis or thermotolerance in either 833K (testis) or HT1376 (bladder) cells (data not shown). Increasing the exposure to 1 h, however, which reduces CFE by 15% for 833K but has no effect on that of HT1376 (Figure 1a), did induce thermotolerance in both cell types. As indicated in Figure 7, although the peak level of thermotolerance induced in both cell types is similar, the onset of thermotolerance in HT1376 starts earlier, peaks earlier and lasts longer. However, if the reduction in colony-forming efficiency produced by the priming heat shock in 833K is also taken into account, the percentage increase in survival induced in the testis cells is greater than that observed in HT1376. The same pattern was observed when a priming heat shock of 42°C for 2 h was used (data not shown).

### Discussion

In contrast to most other types of cancer, metastatic testis tumours can be cured using cisplatin-based combination chemotherapy. This differential sensitivity is retained *in vitro*, indicating that it is due to biochemical differences inherent to these cells (Walker *et al*., 1987; Masters *et al*., 1993). In this study we have shown that testis tumour cells are also more sensitive to heat. To study the molecular basis of this differential sensitivity, we have explored the possibility that it is related to the expression of heat shock proteins.

We investigated if the differential heat sensitivity of testis and bladder cancer cells might be related to levels of constitutively expressed HSP 90, 73, 72 and 27. There was no correlation between constitutive levels of HSP 90, 73 or 72 and heat resistance. Levels of HSP 27, however, were much lower in the testis tumour cells. The correlation between high constitutive levels of this HSP and heat resistance in other

**Figure 4** Western blot showing accumulated levels of HSPs in 833K and HT1376 at various times after a heat shock of 42°C for 2 h. Cell lysates (50 000 cells) were separated by SDS-PAGE, using a 12.5% resolving gel. Numbers at the top of the autoradiogram indicate the time (in hours) after heat shock that the lysate was prepared. Numbers underneath each signal represent the fold increase in HSP levels above the control value (C). HSPs were detected using monoclonal antibodies and a peroxidase-labelled secondary antibody.

**Figure 5** Western blot indicating accumulated levels of HSPs in 833K and HT1376 at various times after a heat shock of 45°C for 15 min. Cell lysates (50 000 cells) were separated by SDS-PAGE, using a 12.5% resolving gel. Numbers at the top of the autoradiogram indicate the time (in hours) after heat shock that the lysate was prepared. Numbers underneath each signal represent the fold increase in HSP levels above the control value (C). HSPs were detected using monoclonal antibodies and a peroxidase-labelled secondary antibody.

**Figure 6** Protein synthesis and accumulated levels of HSP in 833K and HT1376 at various times after a heat shock of 45°C for 1 h. (**a**) Autoradiogram showing protein synthesis. Newly synthesised proteins were labelled by incubating cells in [$^{35}$S]methionine for 1 h. Cell lysates (50 000 cells) were separated by SDS-PAGE, using a 12.5% resolving gel. Numbers at the top of the autoradiogram indicate the time after heat shock that cells were labelled, i.e. 0 = 0–1 h, 1 = 1–2 h, 2 = 2–3 h, etc. C = control, non-heat-shocked cells. Numbers on the left-hand side indicate the molecular weight of marker proteins in kDa. (**b**) Western blot showing accumulated levels of HSPs for samples prepared in **a**. Cell lysates (50 000 cells) were separated by SDS-PAGE, using a 12.5% resolving gel. Numbers at the top of the autoradiogram indicate the time (in hours) after heat shock that the lysate was prepared. Numbers underneath each signal represent the fold increase in HSP levels above the control value (C). HSPs were detected using monoclonal antibodies and a peroxidase-labelled secondary antibody.

**Figure 7** Induction of thermotolerance in 833K (○) and HT1376 (■), determined by clonogenic assay. Cells were subjected to a priming heat treatment of 42°C for 1 h to induce heat shock protein production, returned to 36.5°C for various periods of time, and then given a second heat shock of 45°C for 15 min (833K) or 45°C for 1 h (HT1376). The increase in colony-forming efficiency of the cells given a priming heat treatment was compared with that of untreated controls. The survival of the control cells is taken as 100% and thermotolerance is demonstrated by the percentage increase above the control value. Points represent the mean of values obtained from three assays. All standard deviations were ± 13 or lower.

cells (Cretien and Landry, 1988; Landry *et al.*, 1989; Crete and Landry, 1990, Lavoie *et al.*, 1993) suggests that the relatively low levels of constitutive HSP 27 in testis cells may contribute to their differential sensitivity to heat, and perhaps chemotherapeutic drugs. We are now testing this hypothesis by overexpressing HSP 27 in testis tumour cells using an expression vector containing the gene for HSP 27. There was no correlation between HSP 27 levels and heat sensitivity in the different bladder cell lines. This may indicate that a critical level of HSP 27 is required, or that other factors govern the relative resistance to heat and drugs of these cells.

When HSP synthesis in response to equitoxic heat shocks is considered, the magnitude of the response produced by the testis cells was less than that of the bladder cells, at least as far as synthesis of HSP 73/72 and HSP 27 is concerned. However, when the same (equidose) heat shock was given to testis and bladder cancer cells, the stress response mounted by 833K was similar to or greater than that of HT1376; despite this, many more testis tumour cells die. These results suggest that, although 833K can respond to a heat stress, constitutive and induced HSP in 833K might be less capable of protecting these cells from heat-induced damage than those in HT1376. Thus, to measure the degree of protection afforded by these HSP, we compared the ability of the cells to develop thermotolerance. Both cell lines developed thermotolerance and the amount obtained was similar for both cell types (Figure 7). This suggests that HSPs in heat-primed testis cells are functionally similar to those in heat-primed bladder cells.

Several studies have demonstrated a good correlation between the development and decay of thermotolerance and HSP synthesis (Lindquist and Craig, 1988; Hahn and Li, 1990; Li and Werb, 1990; Welch, 1990). The development of thermotolerance (Figure 7) and the increase in accumulated levels of any of the HSPs investigated (Western blotting experiments; Figures 3 and 4) were not coincident. In both cell types the maximum increase in levels of HSP 90, HSP 73/72 and HSP 27 occurred well before the peak in thermotolerance. However, these findings do not preclude a role for HSPs in the development of thermotolerance in these cells since, as discussed by Kampinga (1993), the distribution, concentration and localisation of HSPs to critical sites in the cell may influence the acquisition of thermotolerance. To investigate this possibility, we need to investigate the localisation of HSPs in these cell lines before and after heat shock.

In conclusion, our results suggest that constitutive HSP in testis cancer cells do not protect these cells from the adverse effects of a heat shock to the same extent as HSP in bladder cancer cells. In view of the evidence indicating that HSP 70 (Li, 1985; Ciocca *et al.*, 1992) and HSP 27 (Ciocca *et al.*, 1992; Huot *et al.*, 1990, 1991; Oesterreich *et al.*, 1993) contribute to drug resistance in some cells, it is tempting to speculate that the low levels of HSP 27 in the testis cells might also contribute to their drug sensitivity. We have shown that a number of other factors might contribute to the sensitivity of testis tumour cells, including DNA repair capacity (Bedford *et al.*, 1988) and topoisomerase II levels (Fry *et al.*, 1991). We now plan to determine whether HSP 27 and these other pathways interact and their possible influence on the response to chemotherapy of testis tumours in the clinic.

**Abbreviations**

HSP, heat shock protein; SDS, sodium dodecylsulphate.

**Acknowledgements**

This work was supported by the Cancer Research Campaign Grant Number SP1997/0201.

**References**

BEDFORD P, FICHTINGER-SCHEPMAN AMJ, SHELLARD SA, WALKER MC, MASTERS JRW AND HILL BT. (1988). Differential repair of platinum-DNA adducts in human bladder and testicular tumor continuous cell lines. *Cancer Res.*, **48**, 3019-3024.

BRONSON DL, ANDREWS PW, SOLTER D, CERVENKA J, LANG PH AND FRALEY EE. (1980). Cell line derived from a metastasis of a human testicular germ cell tumor. *Cancer Res.*, **40**, 2500-2506.

BUBENIK J, BARESOVA M, VIKLICKY V, JAKOUBKOVA J, SAINEROVA H AND DONNER J. (1973). Established cell line of urinary bladder carcinoma (T24) containing tumour-specific antigen. *Int. J. Cancer*, **11**, 765-773.

CIOCCA DR, FUQUA SAW, LOCK-LIM S, TOFT DO, WELCH WJ AND McGUIRE WL. (1992). Response of human breast cancer cells to heat shock and chemotherapeutic drugs. *Cancer Res.*, **52**, 3648-3654.

CRAIG EA. (1985). The heat shock response. *Rev. Biochem.*, **18**, 239-280.

CRETE P AND LANDRY J. (1990). Induction of HSP 27 phosphorylation and thermoresistance in Chinese hamster cells by arsenite, cyclohexamide, A231187, and EGTA. *Radiat. Res.*, **121**, 320-327.

CRETIEN P AND LANDRY J. (1988). Enhanced constitutive expression of the 27-kDa heat shock protein in heat-resistant variants from Chinese hamster cells. *J. Cell Physiol.* **137**, 157-166.

FRY AM, CHRESTA CM, DAVIES SM, WALKER MC, HARRIS AL, HARTLEY JA, MASTERS JRW AND HICKSON ID. (1991). Relationship between topoisomerase II level and chemosensitivity in human tumor cell lines. *Cancer Res.*, **51**, 6592-6595.

HAHN G AND LI G. (1990). Thermotolerance, thermoresistance and thermosensitisation. In *Stress Proteins In Biology And Medicine*. Morimoto RI, Tissieres A and Georgopoulos C. (eds) pp. 79-100. CSH Press: Cold Spring Harbor, NY.

HUOT J, LAMBERT H, ROY G AND LANDRY J. (1990). Atypical multidrug resistance conferred by expression of the HSP 27 heat shock gene in Chinese hamster cells. *Eur. J. Pharmacol.* **183**, 1632-1633.

HUOT J, ROY G, LAMBERT H, CRETIEN P AND LANDRY J. (1991). Increased survival after treatments with anticancer agents of Chinese hamster cells expressing the human $M_r$ 27,000 heat shock protein. *Cancer Res.* **51**, 5245-5252.

KAMPINGA HH. (1993). Thermotolerance in mammalian cells. Protein denaturation and aggregation, and stress proteins. *J. Cell Sci.*, **104**, 11-17.

LANDRY J, CRETIEN P, LAMBERT H, HICKEY E AND WEBER LA. (1989). Heat shock resistance conferred by expression of the human HSP 27 gene in rodent cells. *J. Cell Biol.*, **109**, 7-15.

LAVOIE JN, GINGRASS-BRETON G, TANGUAY RM AND LANDRY J. (1993). Induction of Chinese hamster HSP 27 gene expression in mouse cells confers resistance to heat shock. HSP 27 stabilisation of the microfilament organisation. *J. Biol. Chem.*, **268**, 3420-3429.

LEE WC, LIN K-Y, CHEN K-D AND LAI KY. (1992). Induction of HSP 70 is associated with vincristine resistance in heat-shocked 9L rat brain tumour cells. *Br. J. Cancer*, **66**, 653-659.

LI GC. (1985). Elevated levels of 70,000 dalton heat shock protein in transiently thermotolerant Chinese hamster fibroblasts and in their stable heat resistant variants. *Int. J. Radiat. Oncol. Biol. Phys.*, **11**, 165-177.

LI GC AND WERB WJ. (1990). Correlation between synthesis of heat shock proteins and development of thermotolerance in Chinese hamster fibroblasts. *Proc. Natl Acad. Sci. USA*, **79**, 3218-3222.

LINDQUIST S AND CRAIG EA. (1988). The heat shock proteins. *Annu. Rev. Genet.*, **22**, 631-677.

LOWER J, LOWER R, STEGMAN J, FRANK H AND KURTH R. (1981). Retrovirus particle production in three of four human teratocarcinoma cell lines. In *Haematology and Blood Transfusion*, Neth R, Gallo RC, Graf T, Mannweilen K and Winkler K. (eds) Vol. 26, pp. 541-544. Springer: Berlin.

MASTERS JRW, HEPBURN PJ, WALKER L, HIGHMAN WJ, TREJDOSIEWICZ LK, POVEY S, PARKAR M, HILL BT, RIDDLE PR AND FRANKS LM. (1986). Tissue culture model of transitional cell carcinoma: characterisation of twenty-two human urothelial cell lines. *Cancer Res.*, **46**, 3630-3636.

MASTERS JRW, OSBORNE EJ, WALKER MC AND PARRIS CN. (1993). Hypersensitivity of human testis tumour cell lines to chemotherapeutic drugs. *Int. J. Cancer*, **53**, 340-346.

MORIMOTO RI, TISSIERES A AND GEORGOPOULOS C. (eds). (1990). *Stress Proteins In Biology And Medicine*. CSH Press: Cold Spring Harbor, NY.

OESTERREICH S, WENG C-N, QUI M, HILSENBECK G, OSBORNE CK AND FUQUA SAW. (1993). The small heat shock protein hsp 27 is correlated with growth and drug resistance in human breast cancer cell lines. *Cancer Res.*, **53**, 4443–4448.

PECKHAM MJ. (1988). Testicular cancer. *Rev. Oncol.*, **1**, 439–445.

PERA MF, FRIEDLOS F, MILLS J AND ROBERTS JJ. (1987). Inherent sensitivity of cultured human embryonal carcinoma cells to adducts of *cis*-diammine-dichloroplatinum(II) on DNA. *Cancer Res.*, **47**, 6810–6813.

RASHEED S, GARDNER MB, RONGEY RW, NELSON-REES WA AND ARNSTEIN P. (1977). Human bladder carcinoma: characterisation of two new tumor cell lines and search for tumor viruses. *J. Natl Cancer Inst.*, **58**, 881–890.

RICHARDS EH, HICKMAN JA AND MASTERS JRW. (1995). Heat shock proteins and cellular drug resistance. *Curr. Perspec. Mol. Oncol.* (in press).

SCHLESINGER MJ, SANTORO MG AND GARCIA E. (eds.). (1990). *Stress Proteins. Induction and Function*. Springer: Berlin.

SEIDMAN AD AND SCHER HI. (1991). The evolving role of chemotherapy for muscle infiltrating bladder cancer. *Semin. Oncol.*, **18**, 585–595.

TANNOCK I, GOSPODAROWICZ M, CONNOLLY J AND JEWETT M. (1989). M-VAC (methotrexate, vinblastine, doxorubicin and cisplatin) chemotherapy for transitional cell carcinomas: the Princess Margaret Hospital experience. *J. Urol.*, **142**, 289–292.

WALKER MC. (1990). Inherent sensitivity and acquired resistance in human testicular germ cell tumours *in vitro*. PhD Thesis, University of London.

WALKER MC, PARRIS CN AND MASTERS JRW. (1987). Differential sensitivities of human testicular and bladder tumor cell lines to chemotherapeutic drugs. *J. Natl Cancer Inst.*, **79**, 213–216.

WELCH WJ. (1990). The mammalian stress response: cell physiology and biochemistry of stress proteins. In *Stress Proteins In Biology And Medicine*, Morimoto RI, Tissieres A, Georgopoulos C. (eds.) pp. 223–278. CSH Press: Cold Spring Harbor, NY.

WELCH WJ. (1993). Mammalian stress response; cell physiology, structure/function of stress proteins, and implications for medicine and disease. *Physiol. Rev.*, **72**, 1063–1081.